# Mouse RAGE Variant 4 Is a Dominant Membrane Receptor that Does Not Shed to Generate Soluble RAGE

**DOI:** 10.1371/journal.pone.0153657

**Published:** 2016-09-21

**Authors:** Yunqian Peng, Naftali Horwitz, Edward G. Lakatta, Li Lin

**Affiliations:** Laboratory of Cardiovascular Sciences, National Institute on Aging, NIH, Baltimore, Maryland, United States of America; University of Illinois at Chicago, UNITED STATES

## Abstract

The receptor for advanced glycation end products (RAGE) is a multi-ligand, immunoglobulin-like receptor that has been implicated in aging-associated diseases. Recent studies have demonstrated that both human and murine *Ager* genes undergo extensive alternative splicing that generates multiple putative transcripts encoding different receptor isoforms. Except for the soluble isoform (esRAGE), the majority of putative RAGE isoforms remain unstudied. Profiling of murine *Ager* transcripts showed that variant transcript 4 (mRAGE_v4), the second most abundant transcript in lungs and multiple other tissues, encodes a receptor that lacks nine residues located within the C2 extracellular section close to the trans-membrane domain. We therefore characterized mRAGEV4 isoreceptor in comparison with the full-length mRAGE (mRAGEFL). Although differing in only nine residues, mRAGEFL and mRAGEV4 display very different cellular behaviors. While mRAGEFL undergoes constitutive, extensive shedding in the cell to generate sRAGE, mRAGEV4 hardly sheds. In addition, we found that while mRAGEFL can localize to both the plasma membrane and the endosome, mRAGEV4 is exclusively localized to the plasma membrane. These very different cellular localization patterns suggest that, in addition to their roles in sRAGE production, mRAGEFL and mRAGEV4 may play distinct, spatiotemporal roles in signaling and innate immune responses. Compared to mice, humans do not have the v4 transcript. Although hRAGE, like mRAGEFL, also localizes to the plasma membrane and the endosome, its rate of constitutive shedding is significantly lower. These observations provide valuable information regarding RAGE biology, and serve as a reference by which to create mouse models relating to human diseases.

## Introduction

The receptor for advanced glycation end products (RAGE) is a multi-ligand, pattern recognition receptor that belongs to the immunoglobulin super-family [1]. Signaling of RAGE has been implicated in multiple aging-associated human diseases including cardiovascular diseases, diabetes, and neuropathy [2, 3]. In addition to the full-length receptor, RAGEFL, one identified isoform of RAGE is its soluble form, sRAGE, which lacks the trans-membrane domain and is secreted to the extracellular milieu [4]. RAGE and sRAGE share the entire extracellular portion encompassing the V, C1 and C2 immunoglobulin-like domains. This feature renders sRAGE to function as a decoy that binds ligands and reduces the inflammatory signaling capacity of its membrane-bound counterpart [5–9]. Recent epidemiological studies have associated a low level of circulating sRAGE with various subclinical pathologies, highlighting the physiological significance of generating and maintaining proper sRAGE level [10–15]. A proper level of circulating sRAGE may serve to balance RAGE signaling, and parry tissue inflammation resultant from signaling events [2, 16].

Soluble RAGE can be generated via alternative splicing both in humans (variant 1 isoform) and mice (variant 1 and 3 isoforms) and the product is termed endogenous sRAGE (esRAGE) [17–19]. Several studies have demonstrated that, in addition to alternative splicing, sRAGE can also be generated via cell surface shedding of the membrane-bound RAGE by sheddases ADAM 10 and/or MMP-9[20, 21]. Shedding -generated sRAGE (also dubbed cleaved RAGE, or, cRAGE) and esRAGE share the V/C1/C2 domains and presumably bind the same ligands; the latter has a longer C-terminal sequence due to an alternative splicing-generated translational frame. It is unclear whether the two forms of sRAGE serve different functions *in vivo*.

Hudson and colleagues have demonstrated that both the human and murine RAGE gene, *Ager*, undergo extensive alternative splicing that generates multiple putative transcripts [17, 18]. Although a majority of these putative, alternatively spliced *Ager* transcripts are not a target for nonsense-mediated mRNA decay, it is unclear whether they are indeed translated to functional receptor isoforms *in vivo*. In addition to the relatively well characterized esRAGE, murine transcript variant 20 (mRAGE _v20), which encodes a receptor that contains a truncated intracellular domain (mRAGEΔICD), has been recently characterized by Hudson and colleagues [22]. RAGE_v20 is the most dominant *Ager* transcript in murine kidney (86% of estimated *Ager* transcripts), but not human kidney (4% of estimated *Ager* transcripts). RAGEΔICD is not a dominant isoform in human or murine tissues except for murine kidneys. The authors demonstrated that this specific iosreceptor functions in a dominant negative manner that down-regulates RAGE signaling and affects cell adhesion and viability.

In the current work, we identified a new mRAGE isoform encoded by mRAGE_v4 splice. We showed that while mRAGEFL sheds to generate sRAGE in a time-dependent, constitutive manner in the cell, mRAGEV4 hardly sheds. We also demonstrated that mRAGEV4 is exclusively localized to the plasma membrane, whereas mRAGEFL is localized to both the plasma membrane and endosomal membrane. Compared to mice, humans do not have the corresponding *Ager* v4 transcript, and hRAGE, although it can also undergo shedding when over expressed in the cell, has a much lower constitutive shedding rate than its murine counterpart. These observations suggest that, in addition to alternative splicing, humans and mice may use different mechanisms to generate circulating sRAGE, and may differentially regulate their RAGE signaling in organs and tissues. Identification of the unshed mRAGEV4 isoreceptor may provide new insights into RAGE signaling, and into interplays between RAGE and sRAGE in physiological events. It also provides useful information to interpret studies relevant to humans, and may render a better murine model design for studying RAGE-related human diseases.

## Materials and Methods

### Cloning of mRAGEV4

PCR was used to generate mRAGEV4 using an mRAGEFL plasmid (a gift from Dr. Triantafyllos Chavakis, Dresden University, Germany) as the template. mRAGEV4 lacks exon-9 encoded nine residues (residues 320–328) [18]. To exclude these residues, cDNA fragments encoding residues 1–319 (fragment 1) and 329-stop codon (fragment 2) of mRAGEFL were amplified using Vent DNA polymerase (New England Biolabs) that does not add additional nucleotides to the end of the PCR fragment. The primers for fragment 1 are: forward: 5’-GAAGATCTCCGGATCCCC-3’ (pcDNA 3.1 zeo + sequence), and reverse: 5’ phosphor-TGTGACCCTGATGCTGAC-3’ (mRAGEFL sequence); for fragment 2 are: forward: 5’phspoho-ggctctgtgggtgagtct-3’ (mRAGEFL sequence), and reverse: 5’-GCTCTAGATTACGGTCCCCCGGC-3’ (pcDNA 3.1 zeo+ sequence). Fragment 1 and 2 were then ligated and the ligation mixture was used as the template to re-PCR with the forward primer from fragment 1 and the reverse primer from fragment 2. The re-amplified PCR fragment containing mRAGEV4 was then cloned to pcDNA 3.1 zeo+ vector, and the plasmid was sequenced to confirm the correct mRAGEV4 cDNA sequence.

To generate FLAG-, or mcherry- tagged mRAGE, mRAGEV4, and hRAGE, we subcloned the relevant sequences to an epitope-tagging vector containing RAGE signal peptide sequence and the relative tag, as described previously [23].

### Cell lines and transfection

Human lung adenocarcinoma epithelial cell line A549 (ATCC) and Chinese hamster ovary cells expressing CD14 (CHO-CD14) [24] were used in the studies. A549 cells were cultured in Dulbercco’s modified eagle medium (DMEM, Gibco) containing 10% fetal calf serum (FCS), 1:100 GlutaMax supplement (Gibco) and antibiotics. CHO-CD 14 cell were cultured in RPMI1640 (ATCC) supplemented with 10% FCS and antibiotics.

Transfection of A549 cells was performed with Lipofecamine 3000 (Invitrogen), and CHO-CD14 cells with Lipofectamine 2000 (Invitrogen), according to the manufacturer’s instructions.

### Western blotting, immunoprecipitation, and antibodies

Transfected cells were lysed with ELB buffer (300 mM NaCl, 50 mM Tris-HCL pH7.5, 5 mM EDTA, 0.1% NP-40, 1 mM DTT, 1 mM phenylmethanesulphonyl fluoride, and 1:100 protease inhibitor cocktail) and processed as described previously [25]. A total of 10 μg proteins were resolved with SDS 4–12% precast Bis-Tris gel (Life Technologies). To prepare lung lysates from wild-type and RAGE-null mice, the harvested lungs were rinsed in chilled 1x phosphate buffered saline (PBS) with 1:100 protease inhibitor cocktail (Sigma) and 1 mM phenylmethanesulfonyl fluoride (PMSF) to prevent receptor from shedding, and then minced in chilled RIPA buffer (Pierce) supplemented with the same protease inhibitor cocktail, PMSF, and 1mM DTT. The lysates were then homogenized and rotated at 4°C for 1 h. After filtering through cheesecloth, the cleared lung lysates were then centrifuged with 14,000 rpm at 4°C for 30 min and 15 μg of total proteins were resolved with the same condition as the cell lysates. The studies on mice were conducted according to a protocol approved by the Animal Care and Use Committee of the National Institute on Aging, NIH, and were complied with the Guide for the Care and Use of Laboratory Animals (NIH publication no. 3040–2, revised 1999).

Western blotting was performed as described previously [26]. Rabbit anti-RAGE antibodies (H-300) and anti-β-actin antibodies were purchased from Santa Cruz Biotechnology. Mouse anti-FLAG antibodies (unconjugated or horseradish peroxidase conjugated) were from Sigma. Immunoprecipitation of sRAGE were performed on the media collected from FLAG-RAGE-transfected A549 cells 16 h post-transfection. The medium (1 ml) was briefly centrifuged to discard the detached cells and was incubated with 25 μl protein A/G agarose beads (Santa Cruz) and 1 μl anti-FLAG antibodies at 4°C rotating overnight. The beads were then collected with brief centrifugation and washed in ELB buffer with high salt (1 M NaCl) twice and in regular ELB buffer twice. LDS loading buffer (2 X, Life Technologies) was then added to the beads and the samples were heated at 80°C for 10 min prior to electrophoresis. One third of the total immunoprecipitants were resolved and analyzed in western blotting.

### Confocal microscopy

mRAGEFL and mRAGEV4 were C-terminally tagged with mcherry and either co-transfected with GFP-Rab9 or GFP-Rab11 (both were gifts from Dr. Richard Pagano, Addgene plasmid number 12263 and 12674, respectively), or transfected alone, to A549 cells seeded on glass coverslips. After overnight (16 hours) incubation at 37°C, the coverslips were washed with 1 X PBS. For detection of plasma membrane localization, the cells were incubated with 1 μg/ml of Alexa Fluor 488-conjugated cholera toxin B (Invitrogen) on ice for 20 min, and fixed with 4% paraformaldhyde at room temperature for 15 min. For detection of endosomal localization, cells were permeabilized with 0.2% Triton X-100 for 30 min. Cells on coverslips were then blocked with 1% bovine serum albumin (BSA) for 1 h and mounted with ProLong Gold antifade reagent containing 49-6-diamidino-2-phenylindole (DAPI) (Invitrogen) at room temperature for 48 h shaded from light. For lysosomal localization, mRAGEFL-mcherry and mRAGEV4-mcherry were co-transfected with lysosomal marker LAMP1-GFP (a gift from Dr. Esteban Dell'Angelica, Addgene plasmid # 34831) to serum-starved (6 h) A549 cells. Images were obtained with a Zeiss LSM 710 Meta confocal microscope using a Plan-Neofluar 406/1.3 oil DIC objective.

### Cycloheximide chase

Cycloheximide chase was performed as described [26]. Briefly, 16 h post-transfection, A549 cells were the pre-treated with cycloheximide (10 μg/ml) for 1 h, washed, and re-incubated with the medium-cycloheximide. At each indicated time point, medium was collected for ELISA analyses and cells were lysed and processed for western blot.

### ELISA measurements of sRAGE in cell culture medium and in mouse serum

Mouse RAGE Quantikine ELISA kit (R&D Systems Inc. catalog # MRG00) was used to measure sRAGE concentration in cell culture medium and mouse serum. ELISA was performed according to the manufacturer’s instruction, with correction wavelength set to 540 nm. Medium collected from transfected cells was properly diluted for ELISA analyses. Blood samples were collected from the wild-type and RAGE-null mice (both are of C57B/6 background, aged from 6–32 weeks) via tail snipping (protocol approved by IACUC), and allowed to clot for 2 hours at room temperature. The samples were then centrifuged for 20 min at 2000 x g to obtain serum. Serum (10–50 μl) was directly used for ELISA analyses.

### Statistical analysis

Data was expressed as mean ± standard error of the mean (SEM). Unpaired Student’s *t*-test was used to analyze the data, using GraphPad Prism 6 statistical program. A value of *p* < 0.05 was considered statistically significant.

## Results

### Cloning of mRAGEV4 and detection of the isoreceptor in the lung

We had noticed, from the profiling of murine alternative splices performed by Hudson and colleagues [18], that a specific transcript, mRAGE_v4, is quite abundant in multiple murine tissues. In fact, this splice variant is the second most abundant *Ager* transcripts, next to mRAGE, in the lung, brain, and the heart [18, 22]. The mRAGE_v4 transcript is generated by splicing out the entire exon 9, which encodes nine mainly acidic residues of mRAGE. We therefore hypothesized that mRAGE_4 is likely to be translated *in vivo* and may have functions distinct from that of mRAGEFL. Based on this hypothesis, we proceeded to generate mRAGEV4 by deleting residues 320–328, which correspond to the exon 9 coding sequence.

**Fig 1:**
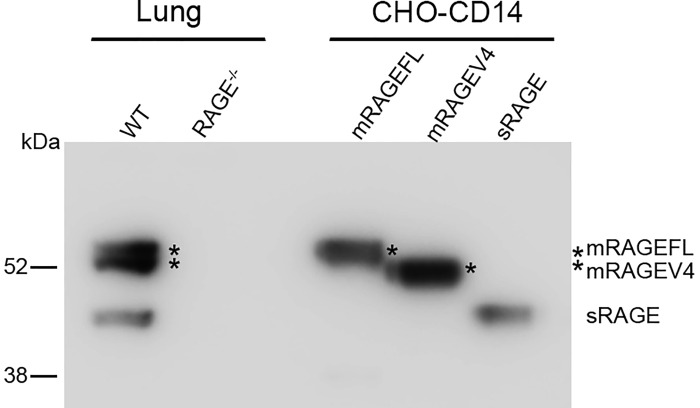
Re-examination of RAGE expression in murine lung. Lung lysates prepared from wild-type (WT) and RAGE^-/-^ mice, and cell lysates from CHO-CD14 cells individually transfected with untagged mRAGEFL, mRAGEV4 and sRAGE cDNA were resolved in SDS 4–12% precast Bis-Tris gel and electrophoresed for 1.5 h. Anti-RAGE antibodies (H-300) were used for western blotting. * marks the resolved mRAGEFL and mRAGEV4.

In previous studies, western blot of lung lysates only detected mRAGEFL and sRAGE, not mRAGEV4 [26], which, given its mRNA abundance, should be detectable in the lung. We reasoned that this might be due to insufficient gel resolution between mRAGEFL and mRAGEV4, as the two receptors differ in only 9 residues. Indeed, studies by Raucci and colleagues showed one additional protein species that is recognized by anti-RAGE antibodies and migrates slightly faster than mRAGEFL in SDS-PAGE [20]. By extending the time for electrophoresis and using individually expressed mRAGEFL, mRAGEV4, and sRAGE as comparisons, we were able to resolve the murine lung lysates such that mRAGEV4 was detected (Fig 1). These results suggest that mRAGEV4 is indeed expressed in murine lung.

### mRAGEV4 does not shed to generate sRAGE

**Fig 2:**
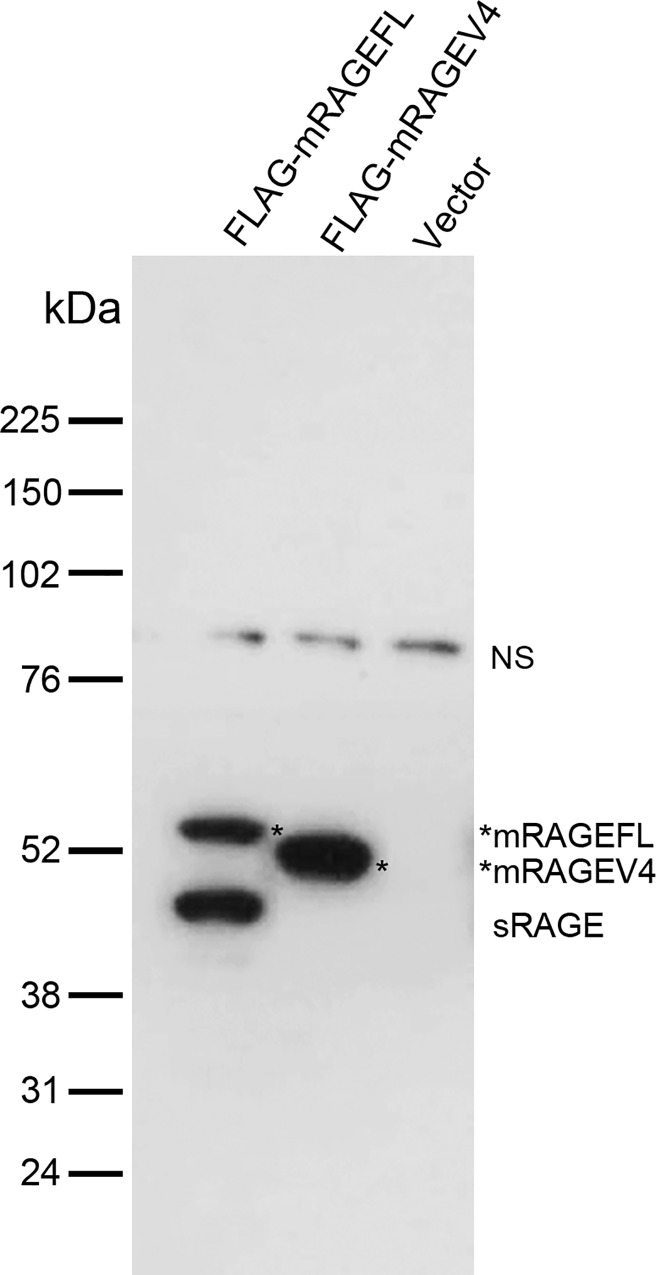
Assessment of mRAGE isoreceptor shedding in the cell. A549 cells were transfected with FLAG-tagged mRAGEFL and mRAGEV4 vectors and cell lysates were prepared 48 h post-transfection and resolved in SDS 4–12% precast Bis-Tris gel. Anti-FLAG antibodies were used for western blotting. * marks the resolved mRAGEFL and mRAGEV4. NS: non-specific, serves as the loading control.

Alveolar type 1 (AT1) cells are known to arise from alveolar type 2 (AT2) cells during natural alveolar renewal or after lung injury [27]. In adult animals, RAGE is richly expressed in alveolar AT1 cells and has been used as a cell type marker for AT1, but its expression in AT2 cells is undetectable [28]. To characterize mRAGEV4 and to compare it with mRAGEFL, we expressed both FLAG-tagged receptors in A549 human lung cells, which are of AT2 origin and do not have detectable endogenous RAGE expression [28]. We observed that mRAGEFL-transfected cells, in a longer post-transfection period (*e*.*g*. 48 h post-transfection), accumulate a significant level of sRAGE that was detectable in cell lysates (Fig 2, lane 1). In contrast, in a short post-transfection period (*e*.*g*. 10–12 h post transfection), only un-cleaved mRAGEFL was detected in cell lysates (Fig 1). Compared to mRAGEFL, mRAGEV4 does not shed to generate sRAGE, although its expression level is comparable to mRAGEFL (Fig 2), suggesting that this isoreceptor is resistant to protease-mediated shedding.

**Fig 3:**
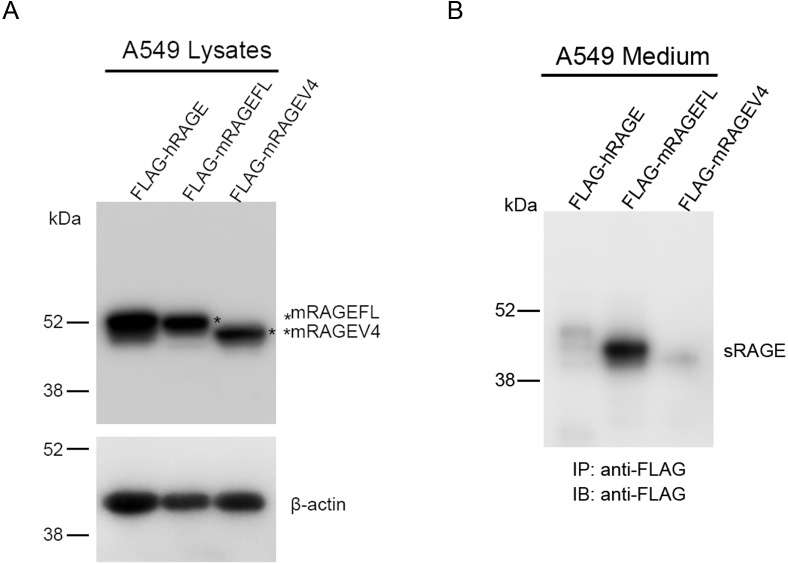
Examination of RAGE shedding using immunoprecipitation. A549 cells were transfected with FLAG-tagged hRAGE, mRAGEFL, and mRAGEV4 expression vectors. Cell culture medium was collected 16 h post-transfection and immunoprcipitated with anti-FLAG antibodies. The cell lysates (A) and immunoprecipitants (B) were resolved with SDS gel and western blotted with anti-FLAG antibodies conjugated with HRP. * marks the resolved mRAGEFL and mRAGEV4, and β-actin level in the cell lysates was used as the loading control for cell lysates.

To assure that mRAGEV4 indeed does not shed to generate sRAGE, and to compare the capacity of sRAGE generation between human and murine RAGEFL via shedding, we tested whether immunoprecipitation can detect the shedding-generated sRAGE in the cell culture medium. A549 cells were transfected with FLAG-tagged RAGE expression plasmids, and cell culture medium was immunoprecipitated with anti-FLAG antibodies. Cell lysates were then resolved with SDS 4–12% Bis-Tris gel for western blot analyses. Although the expression levels of hRAGEFL, mRAGEFL and mRAGEV4 were comparable (Fig 3A), anti-FLAG antibodies detected a high level of sRAGE generated by mRAGEFL in the immuneprecipitants from the cell culture medium, whereas sRAGE was very low in media from hRAGEFL- and mRAGEV4-transfected cells (Fig 3B), suggesting that mRAGEFL undergoes far more extensive constitutive shedding than that of hRAGEFL, or mRAGEV4.

**Fig 4:**
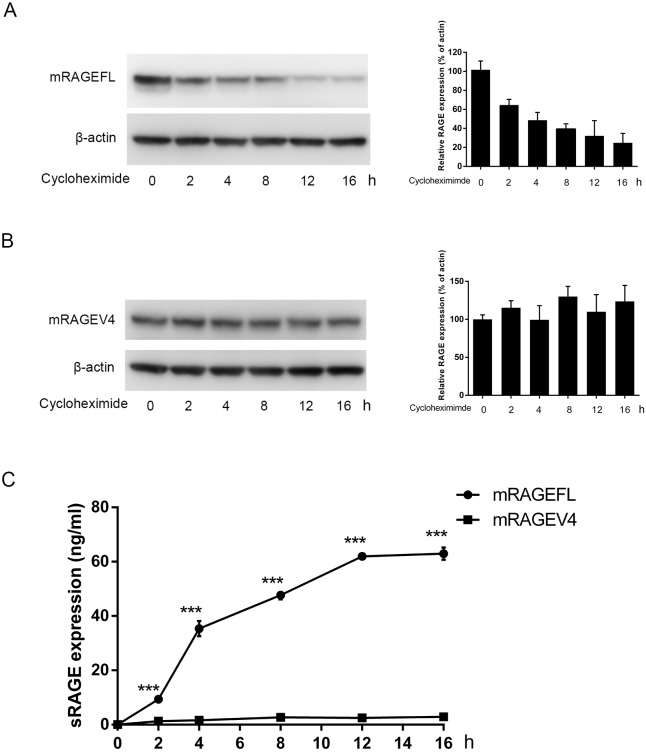
Examination of constitutive RAGE shedding using cycloheximide chase and ELISA analyses. A549 cells transfected with FLAG-tagged mRAGEFL and mRAGEV4 were pre-treated with cycloheximide and then incubated in medium supplemented with cycloheximide. At each time point, cell culture medium (1 ml) was collected for ELISA analyses and cells were lysed for western blotting, using anti-FLAG-antibodies. (A) mRAGEFL; (B) mRAGEV4. For (A) and (B), the left panel is the western blot; the right panel is the densitometry semi-quantification of the western blot. C. ELISA analyses of sRAGE in cell culture medium from mRAGEFL- and mRAGEV4-transfected cells. The ELISA was performed in triplicate, and Student *t*-test was performed to compare sRAGE production between mRAGEFL and mRAGEV4 at each time point. *** *p* < 0.001.

To establish the precursor- product relationship between mRAGEFL and sRAGE, we used cycloheximide chase to study time-dependent shedding of mRAGEFL and to confirm the resistance of mRAGEV4 to shedding. ELISA analysis, which is more sensitive to detect small amounts of target proteins, was employed to detect sRAGE released into the cell culture medium. A549 cells were transfected with FLAG-tagged mRAGEFL and mRAGEV4, and after overnight incubation, the transfected cells were pre-treated with cycloheximide to block *de novo* protein translation, and then chased in cycloheximide-supplemented medium. mRAGEFL displays a shedding pattern that is time-dependent, and releases the generated sRAGE into cell culture medium (Fig 4). Western blot of cell lysates at various time points showed a decrease of mRAGEFL (Fig 4A), and the decrease of mRAGEFL corresponds to the increase of sRAGE in the cell culture medium detected by ELISA (Fig 4C). In stark contrast, the level of mRAGEV4 in cell lysates remains constant during the 16 h cycloheximide chase and very little of sRAGE can be detected in the cell culture medium (Fig 4B and 4C). Together, these results suggest that mRAGEV4 is highly resistant to shedding and that mRAGEFL shedding is likely a major mechanism that contributes to sRAGE in circulation.

### mRAGEV4 and mRAGEFL are differentially localized in the cell

**Fig 5:**
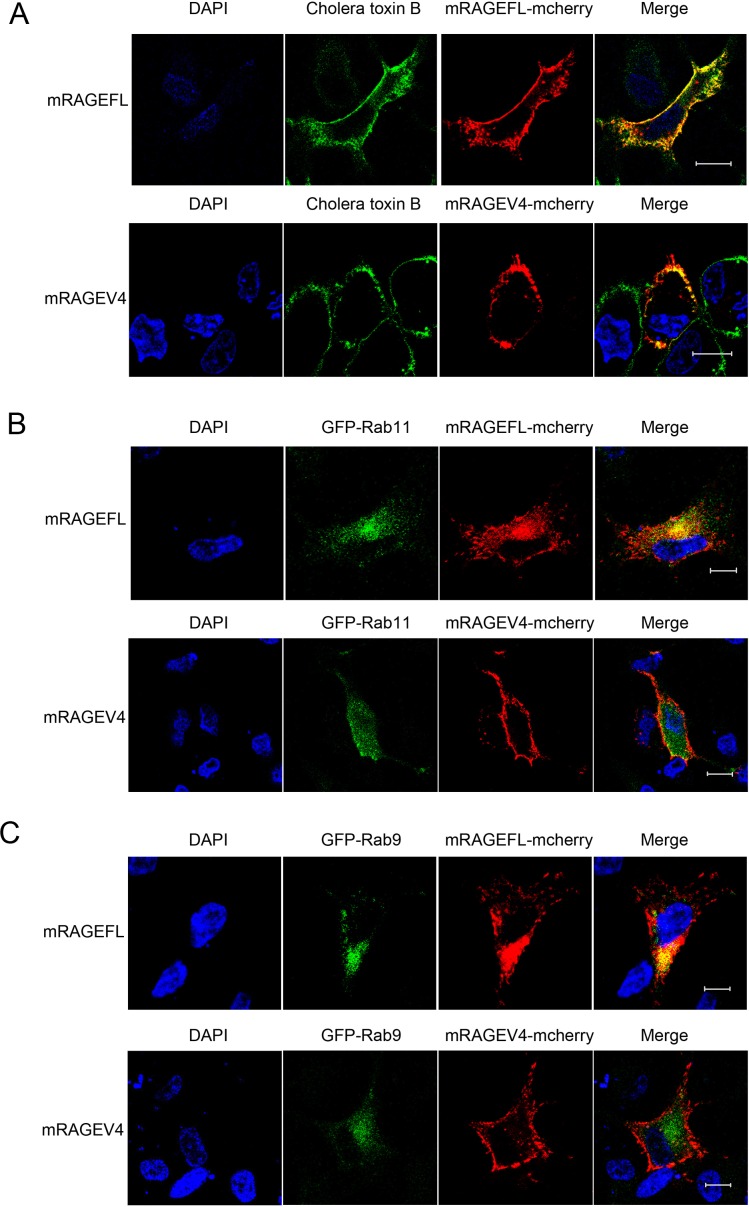
Examination of cellular localizations of mRAGEFL and mRAGEV4. (A) Plasma membrane localization; (B) early endosome localization; (C) late endosome localization. Blue: DAPI (nucleus); red: mcherry (mRAGEFL and mRAGEV4); green: Alexa Fluor 488-conjugated plasma membrane marker cholera toxin B (A), and GFP tagged early endosome marker Rab11 (B) and late endosome marker Rab 9 (C). The scale bar represents 10 μm.

**Fig 6:**
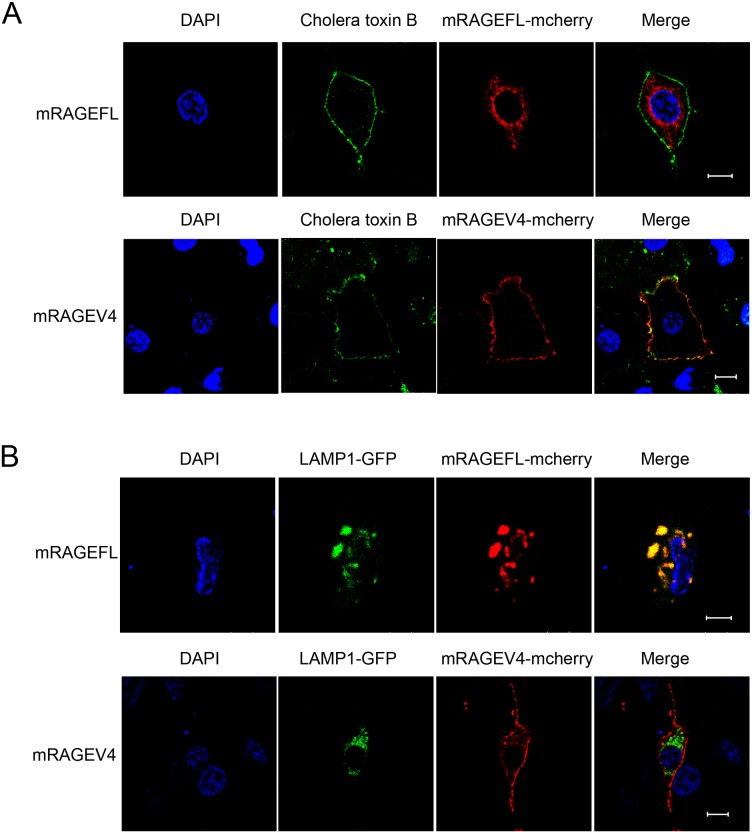
Examination of cellular localization of mRAGEFL and mRAGEV4 in serum-starved A549 cells. A549 cells were serum-starved for 6 h prior to transfection. (A) plasma membrane localization; (B) lysosomal localization. Blue: DAPI (nucleus); red: mcherry (mRAGEFL and mRAGEV4); green: Alexa Fluor 488-conjugated plasma membrane marker cholera toxin B (A), and GFP-tagged lysosome marker LAMP-1(B). The scale bar represents 10 μm.

To further characterize mRAGEV4, we studied its cellular localization in comparison to mRAGEFL. It has been demonstrated that hRAGEFL can be localized to both the plasma membrane and the endosome [23, 29]. Previous studies also showed that in mice, the full-length RAGE, upon binding of pathogen DNA, is internalized to the endosome, whereby it dimerizes with Toll-like receptor 9 (TLR9) and mediates inflammatory signaling [30, 31]. We therefore performed confocal microscopy of A549 cells transfected with mCherry-tagged mRAGEFL and mRAGEV4. Consistent with previous reports, we found that mRAGEFL is localized to the plasma membrane (Fig 5A), as well as to the early and late endosome (Fig 5B and 5C), as indicated by co-transfected early and late endosomal markers (Rab 11 and Rab 9 respectively). In remarkable contrast, mRAGEV4 is exclusively localized to the plasma membrane (Fig 5A–5C). In addition, we found that mRAGEFL is no longer localized to the plasma membrane in serum starved A549 cells (Fig 6A), but is relocalized to the lysosome (Fig 6B). Remarkably, mRAGEV4, under the same condition, remains localized to the plasma membrane (Fig 6B). These different cellular distribution patterns suggest that the two major isoreceptors, in addition to their roles in sRAGE production, may play distinct, spatiotemporal roles in signaling and innate immune responses.

## Discussion

Systematic profiling has shown that both human and murine *Ager* genes undergo extensive alternative splicing, a process that produces multiple putative RAGE isoforms, in addition to the full-length canonical receptor [17, 18]. The complex alternative splicing pattern of the *Ager* gene suggests a far more intricate biological and physiological role of RAGE in inflammatory signaling, cell migration and adhesion. However, a majority of the putative RAGE isoforms remain uncharacterized and their functions unknown. This is largely due to the low expression of RAGE in most tissues, organs and cell types.

We had noticed from the *Ager* transcript profiling performed by Hudson and colleagues that mRAGE_v4 is the second most abundant *Ager* transcript not only in the lung, but also in the brain and heart. In fact, in 1998, Giron and colleagues identified the exact corresponding transcript that skips the coding sequence of residues 320–328 in the liver and kidneys of streptozotocin-induced diabetic rats [32]. Recent RNA-seq analyses performed by Raucci and colleagues further confirmed the existence of mRAGE_v4 transcripts in murine lung (ref). These observations suggest that mRAGEV4 is likely to express in multiple rodent tissues due to its distinct biological functions. Previous studies showed a fast-moving protein species in murine lung tissue that is recognized by antibodies to the N-terminal, but not C-terminal portion of RAGE, suggesting the presence of an additional RAGE isoform in the lung [20]. Careful analyses of murine lung lysates confirmed the previous observation and suggested that the faster moving RAGE species is likely to be mRAGEV4 (Fig 1).

Expressing mRAGEV4 in the cell clearly demonstrated that one striking feature of this RAGE isoform, in comparison to the canonical mRAGEFL, is its resistance to constitutive shedding (Figs 2–4). Shedding of mRAGEFL also appears to be associated with its expression level. An elongated post-transfection incubation causes the cells to produce larger amounts of membrane-bound sRAGE that was detected directly by western blot of cell lysates. mRAGEV4, under the same condition, did not produce detectable sRAGE (Fig 2). When the expression level is low, sRAGE generated by shedding is released into the cell culture medium. Using more sensitive experimental approaches such as immunoprecipitation and ELISA analyses, we also detected shedding-generated sRAGE in the cell culture medium (Figs 3 and 4). These approaches, however, detected only negligible amounts of sRAGE from mRAGEV4 transfected cells (Figs 3 and 4). In particular, while mRAGEFL displays time-dependent, constitutive shedding that releases sRAGE to the medium (Fig 4A and 4C), mRAGEV4 appears to be remarkably stable in the cell during 16 h cycloheximide chase (Fig 4B and 4C). These results suggest that mRAGEV4, in contrast to the canonical mRAGEFL, is resistant to shedding.

In mice, shedding of membrane-bound, full-length RAGE is likely to be the major mechanism of sRAGE generation, as the percentage of esRAGE-coding variants mRAGE_v1 and mRAGE_v3 among total *Ager* transcripts in various tissues is rather low [18]. Consistent with this notion, sRAGE purified from murine lung showed a complete homology with mRAGE ectodomain with a truncated C-terminus ending at residue 329. This analysis suggests that the purified product is generated via proteolytic cleavage, rather than alternative splicing [33]. In contrast to a previous study that was unable to detect sRAGE in murine serum using ELISA [18], we found that circulating sRAGE is readily detectable (S1 Fig). Our preliminary studies showed that circulating murine sRAGE level (~1000 pg/ml) appears to be higher than that of average normal human adults (range 500–900 pg/ml) [14], and remains largely unchanged during 6–32 weeks of life. One possible reason for the discrepancy is that a different ELISA kit was used in the previous study (R & D System DuoSet kit) versus our choice (R & D System Quantikine kit).

In our studies, we found that the rate of constitutive shedding of hRAGE is rather low compared to mRAGEFL. A significant amount of sRAGE can be detected in cell culture medium from mRAGEFL-expressing A549 cells but not from hRAGE-expressing cells (Fig 3). Previous studies showed that hRAGE can shed to generate sRAGE, especially when the cells were treated with phorbol myristate acetate (PMA) [20], or ionomycin [34]. In addition, activation of G protein coupled-receptors has also been found to enhance hRAGE shedding [35]. These studies suggest that hRAGE may have a lower constitutive shedding compared to mRAGEFL, but can be activated to generate more sRAGE in stressed or inflammatory situations. hRAGE and mRAGEFL share approximately 70% of homology, and the sequences surrounding the exon 9-encoded residues are rather diversified. This feature may contribute to the natural propensity of shedding between hRAGE and mRAGEFL in resting state. Recent studies performed in chronic hypoxic mice showed that mRAGE and sRAGE in the lung is decreased, whereas sRAGE in plasma is increased [36]. These observations imply that *in vivo*, mRAGEFL can also be stimulated to generate more sRAGE into the circulation. In the current study, we have not tested whether mRAGEV4 can be shed when cells are stimulated. Since the aforementioned stimuli are known to enhance activities of sheddases, rather than interact with RAGE [21, 34, 35], it is unlikely that they will render mRAGEV4 to undergo shedding. Indeed, Raucci and colleagues in their recent studies demonstrated that PMA enhances mRAGEFL, but not mRAGEV4, shedding in transfected R3/1 rat epithelial cells and murine embryonic fibroblasts, both cell lines are known to express sheddase ADAM10 (ref). From an evolution standpoint, a higher basal level of sRAGE generated by the constitutive shedding of mRAGEFL may suit rodent species, the likes of which encounter more opportunistic pathogen infections and assaults than humans in the natural environment. A higher basal level of sRAGE in the lung and in circulation can avoid “over-reactions” of the immune system to the assaults, and reduce the risk of developing chronical inflammation.

The second striking feature of the newly identified mRAGEV4 is its exclusive localization to the plasma membrane (Figs 5 and 6). mRAGEFL is not only localized to the plasma membrane, but also to the early, and late endosome (Fig 5A–5C, upper panels). In contrast, mRAGEV4 is localized to plasma membrane only (Fig 5A–5C, lower panels).In post-serum starvation cells, the majority of mRAGEFL is no longer localized to the plasma membrane, but to the lysosome, whereas mRAGEV4, in stark contrast, remains localized to the plasma membrane (Fig 6).

RAGEV4 differs from its canonical counterpart, mRAGEFL, in nine residues that are located in the ecto-C2 region close to the transmembrane domain [18]. The nine-residue structural element not only renders cleavage of mRAGEFL by sheddases, it appears also to direct the intracellular cellular trafficking of the full-length receptor. Alternatively, it is also possible that shedding and the subsequent release of the C-terminal potion of RAGE by γ secretase from the plasma membrane are coupled with the internalization. Previous work has demonstrated that RAGE-DNA complexes are localized to the endosome [30], suggesting that full-length RAGE can be internalized to the endosome. The two processes, *i*.*e*. internalization of the full-length RAGE and shedding-generated RAGE C-terminal portion, may not be mutually exclusive. Receptors internalized to the endosome may be recycled back to the cell surface or delivered to the lysosome for degradation [37]. These processes provide the spatiotemporal control of signaling events and are critical in regulating many aspects of cellular physiology. At current stage, the underlying molecular mechanism that regulates the differential cellular trafficking pattern of the two isoreceptors remains unknown. Lacking antibodies that distinguish mRAGEFL and mRAGEV4 also limits our assessment of their functions *in vivo*. The current crystal structure of RAGE contains only V and C1 Ig domains and the structural studies have emphasized on the ligand binding features of RAGE [30, 38, 39]. Whether deletion of nine residues within the C2 ectodomain influences the overall folding of RAGE and hence affects its cellular behaviors is unclear. Future structural studies of hRAGE, mRAGEFL and mRAGEV4 should bring novel insights into our understanding of the molecular mechanism governing their cellular behaviors and physiological functions.

## Supporting Information

S1 Fig ELISA analyses of sRAGE in murine serum. Serum samples collected from the wild-type and RAGE-null mice (both of C57B/6 background, male, 6–32 weeks, n = 10) were analyzed with an ELISA kit for murine sRAGE. Each sample was measured in triplicate and the results were expressed as mean ± SEM.
(TIF)
